# A Visual–Inertial Pressure Fusion-Based Underwater Simultaneous Localization and Mapping System

**DOI:** 10.3390/s24103207

**Published:** 2024-05-18

**Authors:** Zhufei Lu, Xing Xu, Yihao Luo, Lianghui Ding, Chao Zhou, Jiarong Wang

**Affiliations:** 1 Yichang Testing Technique R&D Institute, Yichang 443003, China; 2 School of Artificial Intelligence and Automation, Huazhong University of Science and Technology, Wuhan 430074, China; 3 Institute of Image Communication and Network Engineering, Shanghai Jiaotong University, Shanghai 200240, China; 4 Unit 92228 of the Chinese People’s Liberation Army, Beijing 100072, China

**Keywords:** underwater SLAM, ORB-SLAM, multi-sensor fusion

## Abstract

Detecting objects, particularly naval mines, on the seafloor is a complex task. In naval mine countermeasures (MCM) operations, sidescan or synthetic aperture sonars have been used to search large areas. However, a single sensor cannot meet the requirements of high-precision autonomous navigation. Based on the ORB-SLAM3-VI framework, we propose ORB-SLAM3-VIP, which integrates a depth sensor, an IMU sensor and an optical sensor. This method integrates the measurements of depth sensors and an IMU sensor into the visual SLAM algorithm through tight coupling, and establishes a multi-sensor fusion SLAM model. Depth constraints are introduced into the process of initialization, scale fine-tuning, tracking and mapping to constrain the position of the sensor in the *z*-axis and improve the accuracy of pose estimation and map scale estimate. The test on seven sets of underwater multi-sensor sequence data in the AQUALOC dataset shows that, compared with ORB-SLAM3-VI, the ORB-SLAM3-VIP system proposed in this paper reduces the scale error in all sequences by up to 41.2%, and reduces the trajectory error by up to 41.2%. The square root has also been reduced by up to 41.6%.

## 1. Introduction

The manufacturing and deployment of mines is relatively cheap, while detecting them is a costly and dangerous effort. Due to its features, the seafloor can obscure the devices from sonars, making it hard to detect bottom mines. These mines usually carry larger warheads and more sophisticated sensors than moored ones, which make them much harder to sweep. To detect these bottom mines, autonomous underwater vehicles (AUVs) are increasingly being employed, which are equipped with a sidescan sonar or a synthetic aperture sonar to carry out survey missions [1]. However, the underwater environment poses a unique challenge to vision-based state estimation. In particular, features caused by suspended particles, blurring, light and color attenuation are not as clear as those on the surface of the water. Therefore, the results from different vision-based state estimation packets show a large number of outliers, leading to inaccurate estimation or even complete tracking loss. In the complex underwater environment, single-sensor equipment, such as sonar or cameras, cannot meet the requirements of high-precision autonomous navigation [2,3]. So, the method of multi-sensor fusion is the best choice [4,5].

At present, ORB-SLAM3-VI [6,7] still has the problems of inaccurate scale estimation and easy loss of tracking after initialization after underwater dataset testing. IMUs (inertial measurement units) and optical sensors are local sensors, which can only allow for local observations. But estimation based on local sensors inevitably has a level of cumulative drift. In contrast, global sensors (such as pressure gauges) can allow for global observations, and the output observation data do not have cumulative drift. However, due to noise and a low observation frequency, they cannot be directly used in SLAM (simultaneous localization and mapping) [8,9]. The combination of these two sensors can improve the positioning accuracy of the detection equipment.

For this reason, we introduced new constraints on the scale factor and *z*-axis position in the optimization; that is, we used a pressure sensor to measure the underwater depth of the robot. By introducing depth constraints, we can obtain better estimates of scale information and location. In this paper, we propose a visual–inertial pressure fusion-based SLAM system based on ORB-SLAM3.

## 2. Data Pretreatment

We took the image acquisition time as the benchmark to conduct soft synchronization for multi-sensor data. The frequency of IMUs is generally higher than that of the camera. The IMU measurement data between the current frame and the previous frame are mapped to the current frame, which ensures that each image has a corresponding IMU measurement value during fusion. However, the frequency of the pressure sensor may be higher or lower than the camera frequency, so the two cases need to be discussed separately.

When the frequency of the pressure sensor is higher than that of the camera, each frame will correspond to multiple depth measurements, and the average of the measured values will be taken as the depth of the current frame; that is, $\tilde{d_{i}}=\frac{1}{M}\sum_{k=0}^{M-1}\tilde{d_{k}}$. At this point, the variance of depth measurement becomes $\sigma_{depth}/\sqrt{M}$. When the frequency of the pressure sensor is lower, for example, the camera frequency in the AQUALOC dataset [10] is 20 Hz and the measurement frequency of the pressure sensor is 5 Hz, and some images have no corresponding pressure measurement. At this point, we believe that the underwater robot is not dynamic; that is, the depth change is not significant. We can use interpolation to estimate the depth of this frame; that is:

(1) $$d_{t_{2}}=\frac{t_{2}-t_{1}}{t_{3}-t_{1}}d_{t_{3}}+\frac{t_{3}-t_{2}}{t_{3}-t_{1}}d_{t_{1}}$$

## 3. Factor Graph Optimization

### 3.1. Problem Description

A factor graph can decompose mathematical problems involving multiple variables into products of multiple functions. In the SLAM problem, the factor graph is used as the probability graph model, and the nodes are composed of random variables and probability distributions. The SLAM system needs to use the maximum a posteriori estimation to solve the random variables through observation.

The function of solving the maximum a posteriori estimation problem through factor graph is as follows:

- The factor graph model can describe the mathematical relationship between random variable nodes and observation error nodes.
- When adding a new node to the factor graph, if we first analyze the impact relationship between it and the other nodes, we can only optimize the variables associated with the new node. But, when the scale of the graph changes to some extent, we need to optimize the whole graph. This optimization strategy reduces unnecessary calculation and improves the optimization speed.

**Figure 1:**
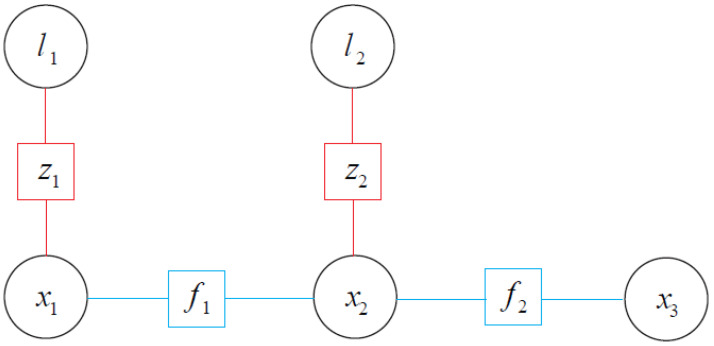
Factor graph of visual SLAM.

For example, the factor graph of a purely visual SLAM can be showed as Figure 1, where *x* is the pose node, *l* is the landmark node, *z* is the observation value and *f* is the equation of motion.

The maximum posterior probability is:

(2) $$\underset{x,l}{\arg max}\prod P\left(x_{k}|x_{k-1}\right)\prod P\left(z_{kj}|x_{k},l_{j}\right)$$

Generally, the conditional probability of each factor is taken as Gaussian distribution. For the equation of motion, it is:

(3) $$x_{k}=f\left(x_{k-1}\right)+w_{k}$$

where $w_{k}∼N\left(0,R_{k}\right)$. So, we can obtain:

(4) $$P\left(x_{k}|x_{k-1}\right)=N\left(f\left(x_{k-1}\right),R_{k}\right)$$

Similarly, for the observation equation, we can obtain:

(5) $$P\left(z_{kj}|x_{k},l_{j}\right)=N\left(h\left(x_{k},l_{j}\right),Q_{kj}\right)$$

where $Q_{kj}$ is the covariance of the noise term of the observation equation, and h is the observation equation.

So, we can solve Formula (10); that is, maximize $N\left(f\left(x_{k-1}\right),R_{k}\right)$ and $N\left(h\left(x_{k},l_{j}\right),Q_{kj}\right)$.

To maximize the Gaussian distribution $x∼N\left(\mu ,\Sigma \right),$ we can minimize its negative logarithm. The negative logarithm of the probability density of the Gaussian distribution is:

(6) $$-ln(P(x))=\frac{1}{2}ln\left(\left(2\pi {)}^{N}det\left(\Sigma \right)\right)\right)+\frac{1}{2}(x-\mu {)}^{T}\Sigma^{-1}(x-\mu )$$

Since the first term is independent of x, we just need to minimize the quadratic term on the right. Combining this with Formula (10), we can obtain:

(7) $$\underset{x,l}{\arg min}\left({\left(x_{k}-f\left(x_{k-1}\right)\right)}^{T}R_{k}^{-1}\left(x_{k}-f\left(x_{k-1}\right)\right)+{\left(z_{k,j}-h\left(x_{k},y_{j}\right)\right)}^{T}Q_{k,j}^{-1}\left(z_{k,j}-h\left(x_{k},l_{j}\right)\right)\right)$$

The error items are defined as:

(8) $$\begin{array}{c} e_{k}=x_{k}-f\left(x_{k-1}\right)\\ e_{kj}=z_{kj}-h\left(x_{k},l_{j}\right) \end{array}$$

For a least-squares problem, $\underset{x,l}{\arg min}{\frac{1}{2}\left\|e\left(x\right)\right\|}^{2}$, we can use the iterative method. First, we give an initial value, and then we can repeatedly calculate the increment to update the optimization variables until the end of the iteration to obtain a better solution. The specific steps are as follows:

1. Give the initial value *x*_0_.
2. In the kth iteration, calculate the increment $\Delta x_{k}$ to minimize ${\left\|e\left(x_{k}+\Delta x_{k}\right)\right\|}^{2}$.
3. When $\Delta x_{k}$ is small enough, stop the iteration; otherwise, solve $x_{k+1}=x_{k}+\Delta x_{k}$ and return to the previous step.

The increment Δx can be obtained by solving the increment equation HΔx=g. In the L-M (Levenberg–Marquardt) method, H is $J^{T}J+\lambda I$.

### 3.2. Graph Optimization Based on g2o

**Figure 2:**
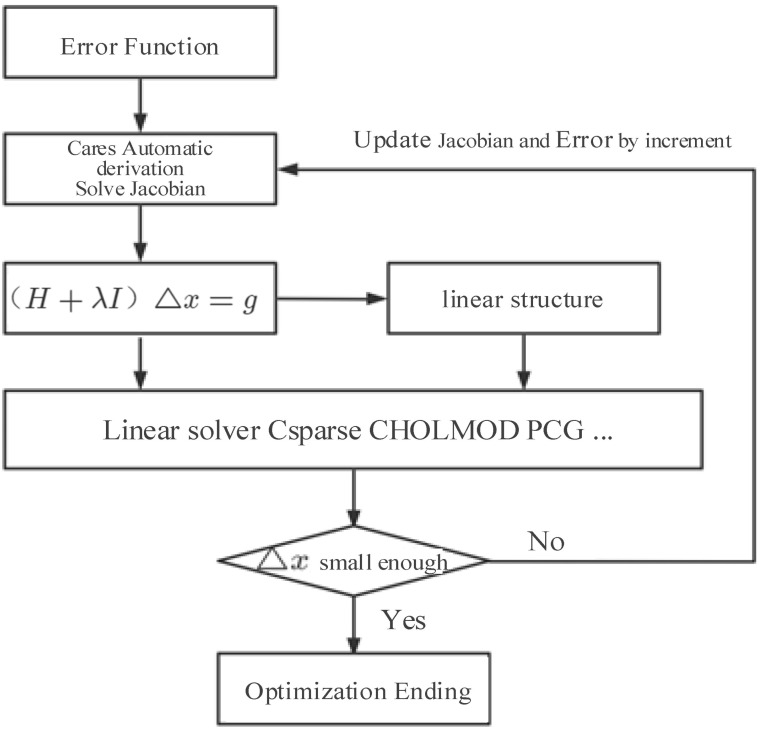
Framework of g2o.

This paper uses the general solver g2o (General Graph Optimization) [11] to solve the graph optimization problem. g2o is a collection of algorithms that can be used to solve the graph optimization problem. It sup ports the use of the definition point method, edge selection method and linear solver to solve nonlinear equations. The algorithm framework is shown in Figure 2.

In practice, we use the Ceres [12] toolkit to calculate the gradient automatically and the Eigen [13] toolkit to solve the linear equation, which is constructed in the form of the L-M method, namely $\left(J^{T}J+\lambda I\right)\Delta x=g$.

## 4. SLAM Algorithm Based on Visual–Inertial Pressure Fusion

**Figure 3:**
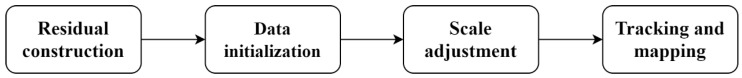
The process of SLAM algorithm based on vision–inertial pressure fusion.

In this section, we tightly couple IMU and pressure measurement with visual data. Because the optimization-based SLAM problem can be expressed by a factor graph, fusing new sensor measurements with the SLAM problem is equivalent to adding new factors and nodes into the graph. The process of our method is shown as Figure 3. Next, we will introduce the specific algorithm process.

### 4.1. Residual Construction

We need to estimate the pose $T_{i}=\left[R_{i},p_{i}\right]$ and velocity $v_{i}$ in the global coordinate system and the gyro and accelerometer deviation, $b_{i}^{g}\mathrm{and}b_{i}^{a}$; the state vector can be calculated using the following formula:

(9) $$S_{i}=\{T_{i},v_{i},b_{i}^{g},b_{i}^{a}\}$$

To solve the state vector, we define the optimization problem as:

(10) $$S_{i}=\underset{S_{i}}{\arg min}\left(E_{\mathrm{visual}\;}\left(S_{i}\right)+E_{\mathrm{imu}\;}\left(S_{i}\right)+E_{\mathrm{depth}\;}\left(S_{i}\right)\right)$$

When there is only a visual sensor, because the gravity direction is unknown, the direction of the *z* axis in the global coordinate system depends on the reference frame of the first frame. It cannot be guaranteed that the *z* axis is parallel to the gravity direction, and the depth information cannot be directly fused. Therefore, we use an IMU to estimate the gravity direction, align the *z* axis of the global coordinate system with the gravity direction, and add the information from the pressure sensor to the optimization constraint. For depth measurement, it can be assumed that the *z* axis of the world coordinate system is collinear with the depth axis, so the depth residual is:

(11) $$S_{i}=\underset{S_{i}}{\arg min}\left(E_{\mathrm{visual}\;}\left(S_{i}\right)+E_{\mathrm{imu}\;}\left(S_{i}\right)+E_{\mathrm{depth}\;}\left(S_{i}\right)\right)$$

The IMU pre-product component rotation, velocity and pose between frame *i* and frame i+1 are expressed as $\Delta R_{i,i+1},\;\Delta v_{i,i+1},\;\Delta p_{i,i+1}$. According to the pre-product component and the state vectors $S_{i}$ and $S_{i+1}$, the construct inertial residuals can be calculated:

(12) $$\begin{array}{l} r_{I_{i,i+1}}=\left[r_{\Delta R_{i,i+1}},r_{\Delta v_{i,i+1}},r_{\Delta p_{i,i+1}}\right]\\ r_{\Delta R_{i,i+1}}=\log\left(\Delta R_{i,i+1}^{T}R_{i}^{T}R_{i+1}\right)\\ r_{\Delta v_{i,i+1}}=R_{i}^{T}\left(v_{i+1}-v_{i}-g\Delta t_{i,i+1}\right)-\Delta v_{i,i+1}\\ r_{\Delta p_{i,i+1}}=R_{i}^{T}\left(p_{j}-p_{i}-v_{i}\Delta t-\frac{1}{2}g\Delta t^{2}\right)-\Delta p_{i,i+1} \end{array}$$

Combined with the visual residual, inertial residual and depth residual, the visual–inertial pressure SLAM can be considered as an optimization problem. Given k + 1 key frames, the state vector $\bar{S_{k}}=\{S_{0}\ldots S_{k}\}$ and *l* 3D points $X=\{x_{0}\ldots x_{l-1}\}$, the optimization problem of visual–inertial pressure can be expressed as:

(13) $$\underset{{\bar{S}}_{k},X}{min}\;\left(\sum_{j=0}^{l-1}\;\sum_{i\in K^{j}}\;\rho_{\mathrm{Hub}\;}\left({∥r_{ij}∥}_{\Sigma_{ij}}\right)+\sum_{i=1}^{k}\;{∥r_{I_{i-1,i}}∥}_{\Sigma_{I_{i-1,i}}}^{2}+\sum_{i=1}^{k}\;{∥d_{i}-d_{0}-p_{z}∥}_{\sigma_{\mathrm{depth}\;}^{2}}^{2}\right)$$

where $K^{j}$ is the set of keyframes in which point *j* can be observed. $\rho_{\mathrm{Hub}\;}$ is the Huber kernel function [14] which can be used to reduce the influence of false matching on the re projection error.

### 4.2. Data Initialization

The purpose of the initialization step is to obtain good initial values for velocity, gravity direction, scale and IMU deviation. Referring to the steps in ORB-SLAM3, the steps of visual–inertial pressure (VIP) fusion initialization are also divided into three steps:

1. Visual estimation. Run the visual SLAM for two seconds, insert keyframes at the speed of 4Hz, and build a map. Using visual residual to constrain pose and map points, obtain initial estimation of pose ${\bar{T}}_{0:k}={\left[R,\bar{p}\right]}_{0:k}$, where the upper dash represents the scaled variable. Because the drift in the visual estimation is very small, it can be used as the constraint of inertial optimization.
2. Inertial pressure estimation. Take the initial estimation of the position and attitude obtained from the previous optimization as the prior of the inertial pressure estimation, and fix the position and attitude ${\bar{T}}_{0:k}$ to optimize the state vector:

(14) $$y_{k}=\left\{s,R_{wg},b,{\bar{v}}_{0:k}\right\}$$

where *s* is the scale factor, which aims to restore the constructed map to the true scale, and $R_{wg}$ is the direction of gravity. The gravity g in the global coordinate system can be expressed as $g=R_{wg}g_{I}$, where $g_{I}={\left(0,0,G\right)}^{T}$, and G is the magnitude of gravity. $b=\left(b^{a},b^{g}\right)\in R^{6}$ represents the deviation in the gyroscope and accelerometer. During initialization, b is regarded as a constant. ${\bar{v}}_{0:k}$ is speed. In the inertial pressure estimation, the pose is not optimized because we think the pose estimated by vision is considered good enough.

**Figure 4:**
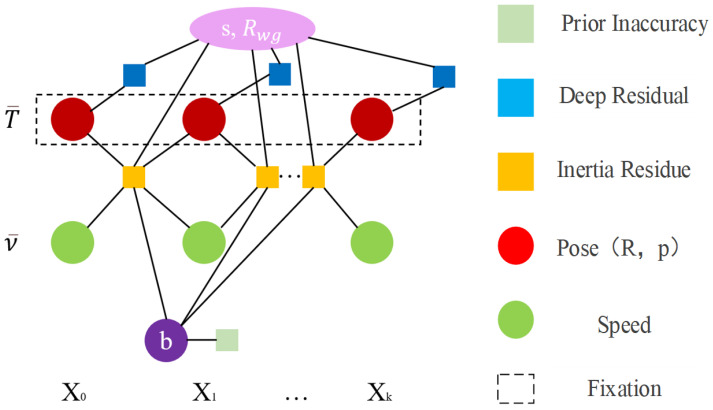
Factor graph representation for inertial pressure optimization.

The problem of inertial pressure optimization can be expressed as:

(15) $$y_{k}^{*}=\underset{y_{k}}{\arg min}\left(∥b{∥}_{\Sigma_{b}}^{2}+\sum_{i=1}^{k}\;{∥r_{I_{i-1,i}}∥}_{\Sigma_{I_{i-1,i}}}^{2}+\sum_{i=1}^{k}\;{∥d_{i}-d_{0}-p_{z}∥}_{\sigma_{\mathrm{depth}\;}^{2}}^{2}\right)$$

where $\Sigma_{b}$ is the prior of IMU deviation, which limits the value range of deviation *b* to ensure that the IMU deviation is closer to 0. A factor diagram of inertial pressure optimization is shown in Figure 4. The vertex of the circle represents the variable to be optimized, and the square represents the factor node/the residual term. $\left\{X_{0},X_{1}\cdots X_{k}\right\}$ represents the k+1 frame keyframe, keyframe $X_{0:k}$ corresponding to pose ${\bar{T}}_{0:k}$ and speed ${\bar{v}}_{0:k}$. *b* is the deviation, which is regarded as a constant in this step and is constrained by a prior deviation. The dotted fixed box indicates that the variable (circular node in the factor graph) is fixed; that is, pose *T* is fixed, and it only participates in the residual calculation as a constraint, and will not be updated in this step. Depth residuals are only used to estimate the state vector $y_{k}$ and gravity direction $R_{wg}$.

After inertial optimization, the map can be scaled according to the estimated proportion, and the global coordinate system of the map can be rotated to align the *z* axis with the estimated gravity direction.

**Figure 5:**
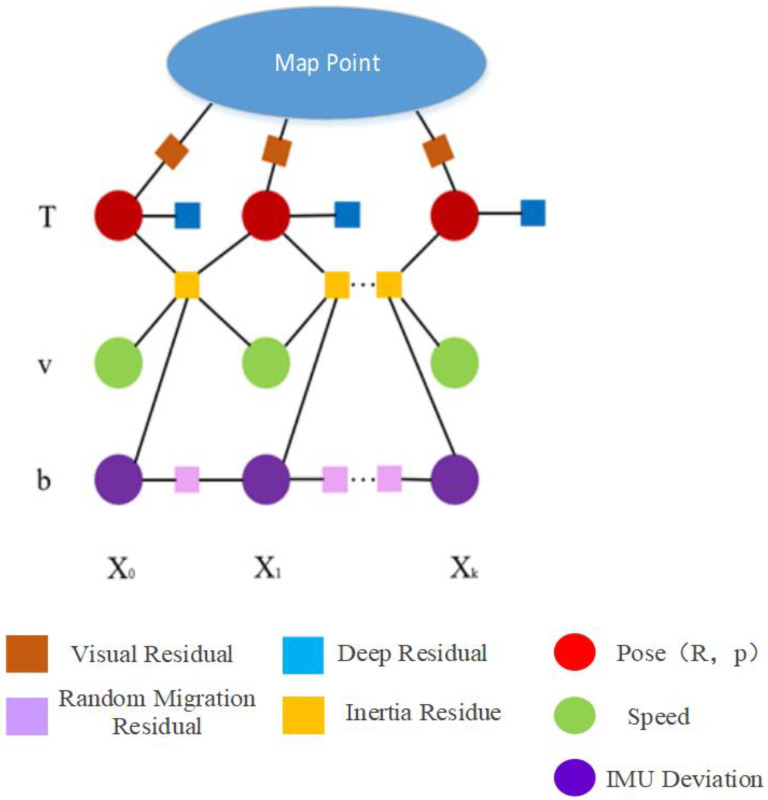
Factor graph representation for Visual-Inertial-Pressure optimization.

3. Visual–inertial pressure joint optimization. Once we have a good estimate of the inertial and visual parameters, we can perform joint visual–inertial pressure optimization to further optimize the state vector and no longer fix the pose and map points. This optimization can be seen in Figure 5. Every frame *X_i_* corresponds to a state vector $S_{i}=\{T_{i},v_{i},b_{i}^{g},b_{i}^{a}\}$; deviation b conforms to random walk model, and the deviation of adjacent frames is constrained by prior random walk residuals. The visual residual constrains the position and key frame pose *T_i_* of the 3D map points. The inertial residual constrains pose *T_i_*, speed *v_i_* and deviation *b_i_*. The depth residual constrains $p_{i}\;$ in pose $T_{i}=\left[R_{i},p_{i}\right]$.

After determining the optimization variables and the corresponding optimization error, we can add the corresponding nodes and edges to g2o to solve the optimization variables.

### 4.3. Scale Adjustment

**Figure 6:**
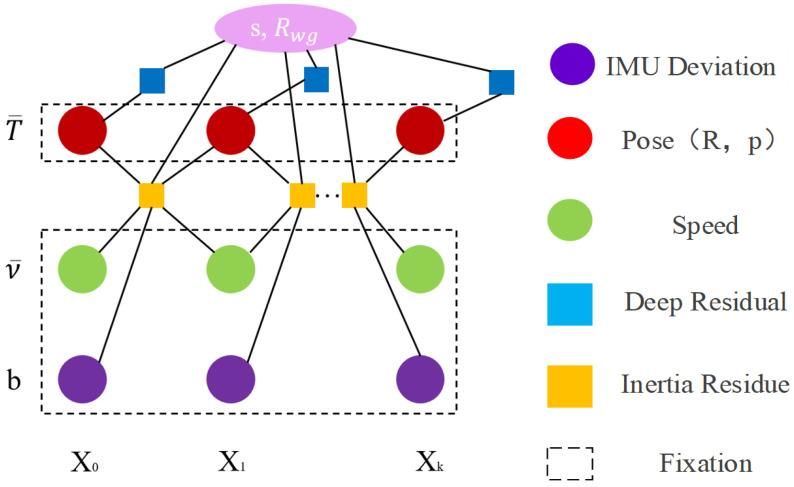
Factor graph representation for scale and gravity optimization.

In some specific cases, such as insufficient robot motion, initialization cannot converge to an accurate solution, so an additional scale adjustment is required. In cluster adjustment, when the scale factor s is explicitly expressed as an optimization variable rather than being implied in T,v, the convergence speed, is much faster [15]. Scale adjustment is based on all the inserted key frames and only adjusts the scale and gravity direction. We do not assume that the IMU deviation is constant and use the estimated deviation of each frame. In the local mapping thread, we run the scale fine adjustment every 10 s until the number of key frames in the map exceeds 200 or initialization has been completed for more than 75 s. A factor diagram of the scale adjustment is shown in Figure 6. The dotted fixed box indicates that the variable (circular node in the factor graph) is fixed; that is, the posture $\bar{T_{i}}$ when the scale is adjusted, speed $\bar{v_{i}}$ and deviation $b_{i}$ are all fixed and they only participate in residual calculation. The scale factor s and gravity direction are optimized, which are constrained by the depth residuals and inertial residuals.

The optimization problem of scale adjustment can be expressed as:

(16) $$\underset{y_{k}}{\arg min}\left(\sum_{i=1}^{k}\;{∥r_{I_{i-1,i}}∥}_{\Sigma_{I_{i-1,i}}}^{2}+\sum_{i=1}^{k}\;{∥d_{i}-d_{0}-p_{z}∥}_{\sigma_{\mathrm{depth}\;}^{2}}^{2}\right)$$

During optimization, the parameters of the incremental update to the gravity direction can be expressed:

(17) $$R_{wg}^{\mathrm{new}\;}=R_{wg}^{\mathrm{old}\;}Exp\left(\delta \alpha_{g},\delta \beta_{g},0\right)$$

where Exp represents the conversion from the rotation vector to the rotation matrix. To ensure that the scale is positive, the update of scale s is expressed as:

(18) $$s^{new}=s^{\mathrm{old}\;}exp(\delta s)$$

**Figure 7:**
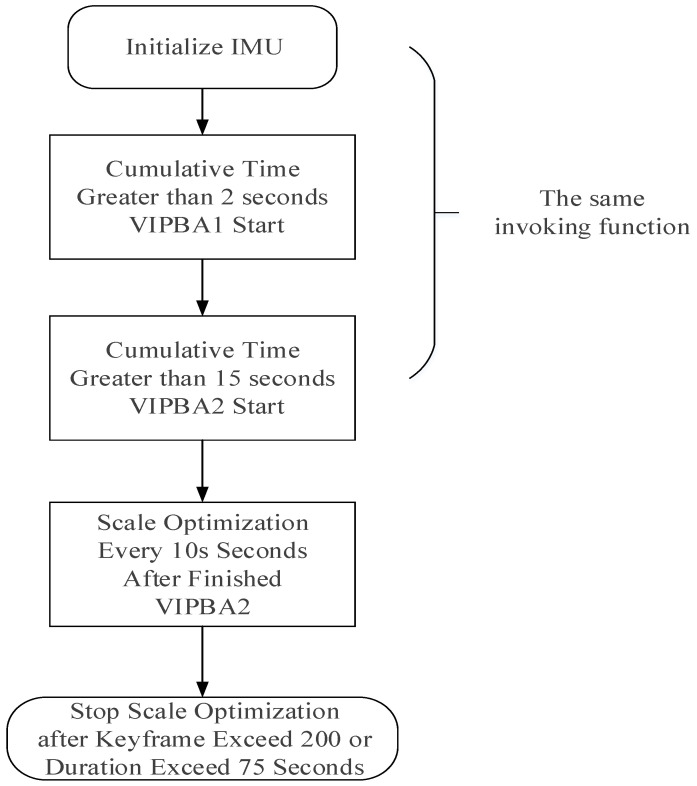
Initialization and scale refinement flow diagram.

In actual use, the local mapping thread has optimized the state vector many times. The initialization and scale fine-tuning processes are shown in Figure 7. IMU initialization, VIPBA1 (Visual Inertial Pressure Bundle Adjustment) and VIPBA2 all call the initialization function Initialize IMU. The initialization function first estimates the IMU state with only inertial pressure constraints, and then optimizes the IMU state with the visual and inertial pressure constraints.

### 4.4. Tracking and Mapping

Visual–inertial pressure tracking is responsible for tracking altitude, speed, IMU deviation and other state variables at the frame rate. The motion model enables us to predict the camera’s attitude in the next frame. After the camera’s altitude is predicted, we can calculate the visual error, IMU error and depth error to optimize the current frame state. Different optimization strategies are used according to whether the map is updated. A map update refers to the generation of new keyframes or the detection of closed loops.

When there is a map update, only the state vector of the current frame is optimized. When the state vector $S_{i}$ is Equation (17), the error is Equation (21). To optimize the error, the optimization result and the calculated matrix H are used prior to the subsequent frames.

When the map is not updated, the state vectors of two adjacent frames are optimized. The corresponding state variable to be optimized becomes $\left\{S_{i},S_{i+1}\right\}$, and the error becomes:

(19) $$E_{\mathrm{visual}\;}\left(S_{i}\right)+E_{\mathrm{visual}\;}\left(S_{i+1}\right)+E_{\mathrm{imu}\;}\left(S_{i},S_{i+1}\right)+E_{\mathrm{depth}\;}\left(S_{i}\right)+E_{\mathrm{depth}\;}\left(S_{i+1}\right)+E_{\mathrm{prior}\;}\left(S_{i}\right)$$

where $E_{prior}$ is the previous error calculated according to the previous frame. Its calculation formula is:

(20) $$E_{\mathrm{prior}\;}\left(S_{i}\right)=\rho \left(\left[e_{R}^{T}e_{v}^{T}e_{p}^{T}e_{b}^{T}\right]\sum_{p}\;{\left[e_{R}^{T}e_{v}^{T}e_{p}^{T}e_{b}^{T}\right]}^{T}\right)$$

where

(21) $$\begin{array}{ll} e_{R}=\log\left({\overset{-}{R}}_{BW}^{i}R_{WB}^{i}\right) & e_{v}={\overset{-}{v}}_{B}-v_{B}^{i}\\ e_{p}={\overset{-}{p}}_{B}^{i}-p_{B}^{i}\; & e_{b}={\overset{-}{b}}^{i}-b^{i} \end{array}$$

ρ is the first-order robust kernel function. In Formula (29), ${\overset{-}{R}}_{BW}^{i},\;{\overset{-}{v}}_{B},\;{\overset{-}{p}}_{B}^{i},\;{\overset{\_}{b}}^{i}$ represent the information about the previous frame, and $R_{WB}^{i},\;v_{B}^{i},\;p_{B}^{i},\;b^{i}$ represent the current optimization result of the previous frame. The purpose of the previous error is to prevent the result of the previous frame from changing too much.

For the local mapping thread, the local window retains N key frames and all points of these key frames, and then optimizes them. At the same time, all other key frames that can observe these points also participate in error calculation, constraining the positions of the map points, but the state of these key frames is fixed in the optimization.

## 5. Results and Evaluation

### 5.1. Scale Optimization Results on Data Set AQUALOC

Multi-sensor fusion can improve the accuracy of SLAM mapping. For monocular SLAM, the most important role of the fusion of other sensors is to restore the true scale.

#### 5.1.1. Scale Error Statistics

We define the scale error according to the length of the predicted track before and after alignment with the real track:

(22) $$\mathrm{Scale}\;\mathrm{error}\;=\frac{\mathrm{Track}\;\mathrm{length}\;\mathrm{before}\;\mathrm{alignment}\;}{\;\mathrm{Track}\;\mathrm{length}\;\mathrm{after}\;\mathrm{alignment}\;}-1$$

We have produced statistics on the scale error in the prediction of trajectory of multiple sequences, and the results are shown in Table 1. A negative scale error means that the predicted map is smaller than the real map, and a positive scale error means that the predicted map is larger than the real map.

It can be seen from the comparison of the results for the scale errors that the scale errors of ORB-SLAM3-VIP’s predicted map tracks are all within 5.4%. Compared with ORB-SLAM3-VI, the scale errors are reduced by 2.6–41.2%. ORB-SLAM3-VI cannot restore the map to the true scale, because the IMU has no direct effect on the position p. It estimates the position p through two integration calculations, which is more vulnerable to noise.

#### 5.1.2. Result Analysis for the Harbor_01 Sequence

Here, we analyze the process of scale optimization. According to the flow chart in Figure 7, the map scale has been adjusted many times in the local mapping thread, including three changes the to initialization functions and n scale adjustments. n depends on the number of key frames being greater than 200 or the cumulative interval time of all key frames in the optimization exceeding 75s. In the process of initialization function and scale adjustment, a scale factor will be calculated. Every time the scale factor is calculated, the size of the map is updated.

**Figure 8:**
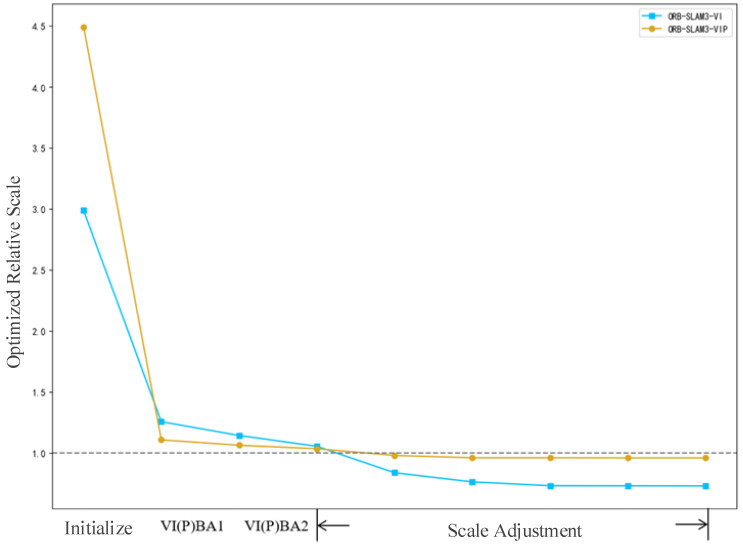
Relative scale of harbor_01 during optimization.

We recorded the scale factor obtained by each optimization when running SLAM on sequence harbor_01, and calculated the length ratio of the predicted trajectory and the predicted trajectory after scale alignment, which is recorded as the relative scale. The scale proportions of predicted trajectory of ORB-SLAM3-VI and ORB-SLAM3-VIP are 0.767 and 0.958, respectively. According to the optimized scale factor, we can deduce the relative scale of each optimized map and draw the image. The change in the relative scale in the optimization process is shown in Figure 8.

If the relative scale in the figure is 1, which means that the scale is the same as the real map. The relative scale before initialization is the scale of the map constructed by the purely visual SLAM relative to the real map, which can be considered as random. It can be seen from Figure 8 that the scale estimation of the VIP system converges faster and more accurately than that of the VI system. It is proved that the proposed vision–inertial pressure fusion framework is effective in restoring the monocular SLAM scale.

**Figure 9:**
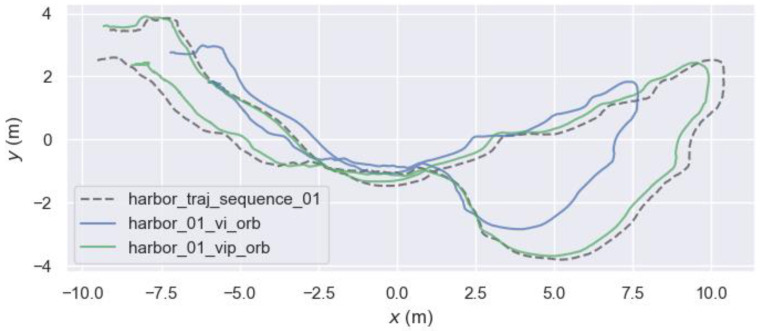
Comparison of scale unaligned estimate trajectory for harbor_01 underwater sequence.

We compared the mapping results of ORB-SLAM3-VI and ORB-SLAM3-VIP without scale alignment, as shown in Figure 9.

**Figure 10:**
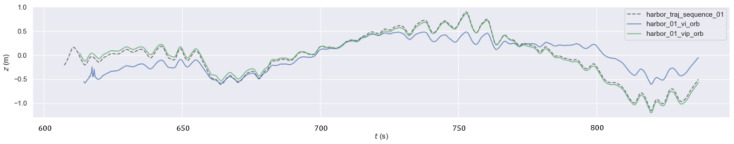
Prediction of the *z*-axis position for harbor_01 sequence.

The prediction of the *z*-axis position by the two algorithms is shown in Figure 10. The z axis direction represents the direction of gravity in the world coordinate system. It can be found that the introduction of pressure sensor makes the depth estimation of the algorithm closer to the true value.

### 5.2. Track Prediction Results on Data Set AQUALOC

In this section, ATE RMSE is also used to evaluate the mapping trajectory, and the scale of the predicted trajectory is aligned with the true value without considering the results of the scale optimization. We conducted experiments to produce statistics for ATE RMSE values of different methods, as shown in Table 2, where VI represents the joint optimization of visual inertia, and VIP represents the joint optimization of visual and inertia pressure. We tested on the AQUALOC dataset and calculated the absolute trajectory error RMSE of each algorithm after scale alignment. The test on the AQUALOC dataset showed that IMU’s estimation of motion is not accurate when the lack of visual constraint is too long, and it is easy to “fly” along the trajectory. After we lose visual tracking on the harbor_04 and harbor_07 sequences, we no longer use the IMU for tracking.

From Table 2, we can see that the RMSE of the VIP system is 6.1–41.6% lower than that of the VI system. It is shown that the introduction of the pressure sensor has improved the mapping accuracy.

## 6. Conclusions

Based on ORB-SLAM-VI, we propose the ORB-SLAM-VIP algorithm for visual–inertial pressure fusion. First, we introduce IMU and pressure sensor models, including the corresponding relationship between pressure and depth, the IMU motion model and IMU pre integration. Then, we introduce how to carry out tight coupling optimization. We give a method to calculate the error term of the IMU and pressure sensors, and introduce the optimization process to the initialization, scale fine-tuning, tracking and mapping steps. Finally, we tested our method on the AQUALOC dataset and evaluated it from the perspective of monocular scale recovery and trajectory error, respectively. The experimental results show that, compared with ORB-SLAM-VI, ORB-SLAM-VIP can not only restore the scale more correctly, but can also improve the accuracy of mapping to a certain extent. The proposed method can be applied to improve the operational efficiency of naval mine countermeasures.

## Figures and Tables

**Table 1:** Absolute trajectory error [16] of harbor sequences.

Algorithm	Harbor_01	Harbor_02	Harbor_03	Harbor_04	Harbor_05	Harbor_06	Harbor_07
ORB-SLAM3-VI	−23.3%	−17.1%	5.0%	−18.3%/43.7%	6.6%	−31.6%	−2.7%/−18.2%
ORB-SLAM3-VIP	−4.2%	−2.6%	−0.8%	3.4%/2.5%	0.4%	5.4%	-0.1%/1.5%

**Table 2:** Absolute trajectory error in harbor sequences.

Sequence		Harbor_01	Harbor_02	Harbor_03	Harbor_04	Harbor_05	Harbor_06	Harbor_07
	RMSE	0.437	0.081	0.108	0.140/0.237	0.076	0.057	0.081/0.203
ORB-SLAM3-VI(m)	min	0.039	0.005	0.009	0.043/0.068	0.012	0.006	0.029/0.065
	max	1.191	0.163	0.239	0.342/0.486	0.156	0.138	0.187/0.374
	RMSE	0.113	0.054	0.051	0.098/0.151	0.047	0.026	0.044/0.108
ORB-SLAM3-VIP(m)	min	0.021	0.005	0.007	0.022/0.039	0.009	0.006	0.019/0.047
	max	1.082	0.157	0.129	0.289/0.427	0.134	0.116	0.130/0.296
UW-VIP(m) [17]	RMSE	0.42	0.37	0.26	1.56	0.09	0.06	1.16

## Data Availability

AQUALOC dataset: http://www.lirmm.fr/aqualoc/ (accessed on 15 May 2024).
